# Segmentectomy quality remains important in ground‐glass‐dominant stage I lung cancer

**DOI:** 10.1111/1759-7714.15162

**Published:** 2023-11-27

**Authors:** Wongi Woo, Jimin Lee, Dae Hyun Jin, Jihoon Kim, Duk Hwan Moon, Sungsoo Lee

**Affiliations:** ^1^ Department of Thoracic and Cardiovascular Surgery, Gangnam Severance Hospital Yonsei University College of Medicine Seoul Republic of Korea

**Keywords:** clinical outcomes, ground‐glass opacity, lung cancer, quality management, segmentectomy

## Abstract

**Background:**

Segmentectomy for early‐stage lung cancer has benefits for survival and parenchymal preservation. However, segmentectomies are technically challenging, thereby resulting in considerable variability in the quality of resection. In this study, we aimed to review the quality of segmentectomies and analyze their clinical impact.

**Methods:**

This retrospective study reviewed patients diagnosed with stage I lung cancer after segmentectomies between 2013 and 2021. Segmentectomies were classified as anatomical or nonanatomical; anatomical resection included segmental bronchus and vessel (artery and/or vein) divisions; others were classified as nonanatomical. The primary outcome was recurrence‐free and overall survival, and the secondary outcome was postoperative spirometry and lung plication, which is seen as a fibrotic line along the stapling site.

**Results:**

Of the 132 segmental resections included in this study, 101 (76.5%) were anatomical segmentectomies. The median consolidation‐tumor ratio was 0.40, and 83.3% (110/132) had ground‐glass opacities (GGOs). Compared to nonanatomical resections, more N1 and total lymph node stations were retrieved after anatomical segmentectomies. Regarding clinical outcomes, recurrence‐free survival was better after anatomical segmentectomy (*p* = 0.049); however, overall survival was not significantly different (*p* = 0.064). Furthermore, at 3–6 months postoperatively, thicker lung plication at the stapling site was observed in nonanatomical resections (*p* < 0.001). Subgroup analysis for complex segmentectomies revealed a larger decrease in forced‐expiration volume in 1 s after nonanatomical resection.

**Conclusion:**

Anatomical segmentectomy resulted in better survival and a lower incidence of thick lung plication, even in GGO‐dominant tumors. Therefore, further standardization and quality management of segmentectomy procedures will improve the clinical outcomes.

## Graphical abstract

Qualified segmentectomy resulted in better survival and a lower incidence of thick lung plication after surgery, even in ground‐glass opacity (GGO)‐dominant tumors.

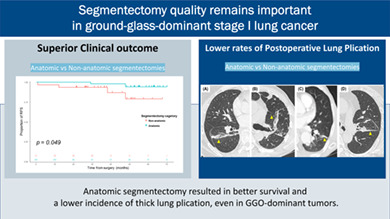

## INTRODUCTION

Lung cancer screening programs and advances in medical therapy have improved the clinical outcomes of patients with lung cancer.^1^, ^2^, ^3^ For these patients, surgery has evolved with the introduction of minimally invasive approaches and rigorous discussion regarding the optimal extent of resection.^4^, ^5^, ^6^ Particularly, lung cancer resections have become much smaller than those in the past, when lobectomy was recommended as the primary choice. Recent randomized clinical trials on sublobar resections have led to the generalized introduction thereof in patients with early‐stage lung cancer.^5^, ^6^

Segmentectomy for lung cancer (size <2 cm) may have better long‐term outcomes than lobectomies and preserves lung volume, thereby allowing patients to have a better functional reserve for future cardiopulmonary events. However, the higher recurrence rate after segmentectomy is concerning. Additionally, segmentectomy procedures are not clearly defined among thoracic surgeons in academic and nonacademic settings.^7^ Owing to the technical difficulty and complexity of segmental structures, appropriate processes must be developed.

Weiss et al.^8^ highlighted the diversity, or irregularity, of segmentectomies with respect to individual procedures. They clarified the definitions of anatomical and nonanatomical segmentectomies and evaluated the quality of segmentectomies at a single institution based on these criteria. Interestingly, 19.2% of segmentectomies were classified as nonanatomical, even in highly academic settings. A National Cancer Database study from the USA also demonstrated a heterogeneous description of segmentectomy, with only 12.6% meeting all quality measures.^7^ This alarmed the surgical society and urged them to develop and evaluate the appropriateness of segmentectomies, including mediastinal lymph node dissection (MLND), optimal surgical candidates, and sufficient resection margins. There is also a significant difference in adherence to quality measures based on hospital setting; many nonacademic institutions perform segmentectomies for wide‐wedge or nonanatomical resections.

Segmentectomy is a complex procedure requiring a certain level of technical skill. Additionally, not all patients can undergo anatomical segmentectomies due to the complexity of segmental anatomy. Moreover, segmental anatomical resection can result in more postoperative complications, such as prolonged air leakage, resulting in a longer hospital stay. Notably, the long‐term oncological effects of anatomical segmentectomy and nonanatomical resection in early‐stage lung cancer are unclear. Therefore, we aimed to evaluate the quality of segmentectomies at our institution and compare the clinical outcomes of patients according to the segmentectomy quality.

## METHODS

### Participants

This retrospective study included patients diagnosed with pathological stage I non‐small cell lung cancer (NSCLC) between October 2013 and December 2021. Of 859 patients, only those who underwent segmentectomies were included (*n* = 150). After excluding patients with stage 0 disease (*n* = 18), 132 patients were included in this study.

### Segmentectomy classification

Surgical charts were reviewed, and the types and number of staplers were used to classify the quality of segmentectomy and the operation records. A previous study reported that anatomical segmentectomy should involve the resected segmental bronchus and at least one vascular structure (segmental artery or vein).^8^ Cases not meeting these criteria were classified as nonanatomical segmentectomies. If there was no specific information regarding the division of the major structures, we reviewed the types and number of staplers to evaluate whether proper vascular division was performed.

### Operative techniques

All surgeries were performed under general anesthesia by three thoracic surgeons, including two junior surgeons. The patients underwent either thoracotomy or video‐assisted thoracoscopic surgery; cases that were converted to thoracotomy due to technical or anatomical difficulties were classified as thoracotomies. Stapling was only used for the division of intersegmental planes, and the identification of intersegmental planes using indocyanine green injection had been used since 2020; before this, intraoperative ventilation was used to distinguish intersegmental planes. Radical lymph node dissection was performed along the anatomical landmarks, and the location and number of stations removed were decided according to the radiological characteristics of the tumor, intraoperative frozen biopsies, and the surgeons' preferences. Segmentectomies were classified according to their level of difficulty: simple segmentectomies included superior segmentectomies (LS6, RS6), left trisegmentectomy (LS1–3), lingular segmentectomies (LS4 + 5), and composite basal segmentectomies (RS7‐10, LS7‐10). All other segmentectomies were regarded as complex segmentectomies due to their technical difficulty.^9^, ^10^, ^11^

### Follow‐up protocols and radiological evaluation

**FIGURE 1:**
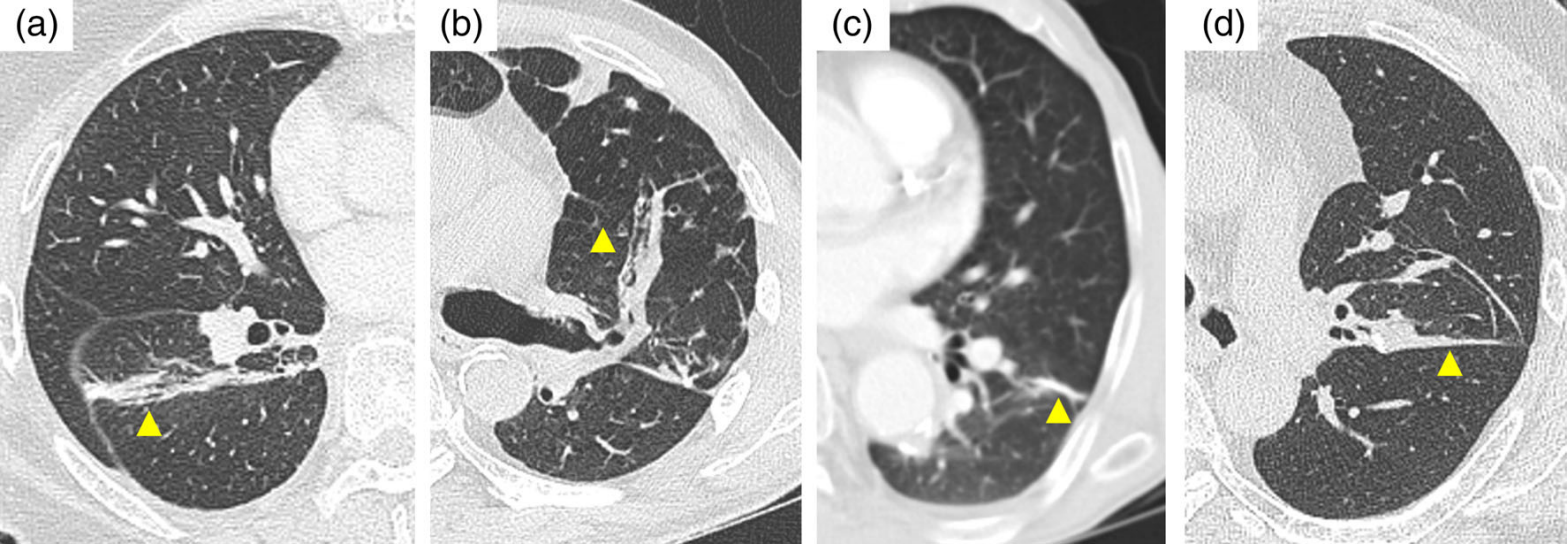
Computed tomography imaging of lung plication after segmentectomy. After nonanatomical segmentectomy, many cases had thicker and wider lung plication, as shown in (a) and (b). (c) and (d) were common findings mostly after anatomical segmentectomy.

Patients were regularly followed up within 2 weeks of postoperative discharge. Thereafter, patients visited the outpatient clinic 1, 3, 6, and 12 months postoperatively. Postoperative computed tomography (CT) was generally performed 3–6 months after the surgery, and axial images were reviewed to evaluate postoperative plication along the stapling lines. Radiopaque linear lines identified in the lung window (−500 Hounsfield units) were classified as lung plications (Figure 1). Discretion between lung plication and possible recurrence or other findings were made from collaboration with radiologists. Thickness was used to classify them into two categories with a 5‐mm cutoff value. Other findings, such as residual hydropneumothorax or pleural effusion, were also included in the radiological imaging review.

### Statistical analysis

For continuous variables, data are presented as medians and interquartile ranges. Mann–Whitney U test was used to analyze continuous variables and Fisher's exact test was used to compare categorical variables. Cox proportional hazard analysis was used to identify relevant risk factors for recurrence‐free and overall survival and survival curves were presented via the Kaplan–Meier log‐rank test. Statistical analyses were performed using R version 4.0.4 (R Core Team, R Foundation for Statistical Computing, Vienna, Austria), and differences were considered statistically significant at a two‐tailed *p*‐value < 0.05.

## RESULTS

### Clinical characteristics of patients

The clinical characteristics of all included patients are presented in Table 1. The cohort included patients with significant ground‐glass opacities (GGOs), and >50% of the patients were female and never smokers. Of the 132 patients, 101 (76.5%) underwent anatomical segmentectomies; the proportion of anatomical segmentectomies has increased since 2019 (*p* = 0.052). There were no differences in the demographics and preoperative radiological characteristics of tumors—including diameter, consolidation tumor ratio, peripheral location, metabolic activity, and preoperative pulmonary function—between the two groups.

**TABLE 1:** Preoperative characteristics of patients according to the quality of segmentectomy.

Factor	Segmentectomy category		*p*‐value
	Nonanatomical	Anatomical	
	*N* = 31	*N* = 101	
Age	62.0 [56.5, 71.5]	63.0 [53.0, 71.0]	0.751
Sex			0.302
Female	16 (51.6)	63 (62.4)	
Male	15 (48.4)	38 (37.6)	
Diabetes mellitus	4 (12.9)	17 (16.8)	0.781
Hypertension	10 (32.3)	38 (37.6)	0.672
Cardiovascular diseases	1 (3.2)	9 (8.9)	0.451
COPD	1 (3.2)	1 (1.0)	0.416
Smoking history			0.205
Never smoker	22 (71.0)	83 (82.2)	
Ex or current smoker	9 (29.0)	18 (17.8)	
Radiological characteristics			
Tumor diameter	13.0 [9.0, 15.0]	13.0 [10.0, 20.0]	0.231
Solid portion diameter	6.0 [1.5, 11.0]	6.0 [1.0, 10.0]	0.972
Consolidation tumor ratio (CTR)	0.40 [0.18, 0.74]	0.40 [0.10, 0.73]	0.808
CTR < 0.25	9 (29.0)	31 (30.7)	0.897
0.25 ≤ CTR < 0.75	14 (45.2)	48 (47.5)	
CTR ≥ 0.75	8 (25.8)	22 (21.8)	
Peripheral location			0.838
Central lesion	14 (45.2)	49 (48.5)	
Peripheral lesion	17 (54.8)	52 (51.5)	
Distance from peripheral visceral pleura, mm	13.0 [3.0, 24.2]	15.0 [10.0, 24.0]	0.472
Metabolic activity on PET scan			0.458
No uptake	18 (58.1)	47 (46.5)	
Mild	5 (16.1)	29 (28.7)	
Hypermetabolic	6 (19.4)	15 (14.9)	
Not done	2 (6.5)	10 (9.9)	
Pulmonary functions and laboratory values			
DLCO, %	100.0 [89.0,110.0]	98.0 [87.5, 109.0]	0.924
FVC, L	3.20 [2.82, 3.95]	3.26 [2.89, 3.93]	0.843
FEV1, L	2.38 [2.10, 2.75]	2.38 [2.12, 2.96]	0.882
FEV1/FVC, %	75.5 [70.0, 82.5]	76.5 [72.0, 79.7]	0.559
Albumin, mg/dL	4.40 [4.20, 4.45]	4.40 [4.20, 4.50]	0.705
Hemoglobin, g/dL	13.4 [12.6, 13.9]	13.4 [12.6, 14.4]	0.753

*Note*: Data are presented as *n* (%) or median [interquartile range].

Abbreviations: COPD, chronic obstructive lung disease; CTR, consolidation to tumor ratio; DLCO, diffusing capacity of the lung for carbon monoxide; FEV1, forced expiratory volume in one second; FVC, forced vital capacity; PET, positron emission tomography.

### Operative results according to the types of segmentectomy

More than half of surgeries (62.8%, 83/132) were performed using VATS, and this did not differ according to the segmentectomy quality (Table 2). Anatomical resection was performed more frequently in simple segmentectomies, such as left trisegmentectomy, and lingular, superior, or complex basal segmentectomies (*p* = 0.007). Regarding MLND, more N1 (*p* < 0.001) and total stations (*p* < 0.001) were retrieved in the anatomical than that in the nonanatomical segmentectomy group; the difference in N2 stations was not statistically significant (*p* = 0.073). Regarding the resection margin, a significantly larger safety margin was obtained with anatomical resection than that with nonanatomical segmentectomy (median, 1.5 vs. 2.0 cm; *p* = 0.009) (Table 2).

**TABLE 2:** Surgical and clinicopathological outcomes according to the quality of segmentectomy.

Factor	Segmentectomy category		*p*‐value
	Nonanatomical	Anatomical	
	*N* = 31	*N* = 101	
Approach			0.532
Thoracotomy	13 (41.9)	36 (35.6)	
VATS	18 (58.1)	65 (64.4)	
Segmentectomy grade			
Simple	11 (35.5)	64 (63.4)	0.007
Complex	20 (64.5)	37 (36.6)	
Period of surgery			
2013–2018	16 (51.6)	31 (30.7)	0.052
2019–2021	15 (48.4)	70 (69.3)	
Number of segments resected	1.0 [1.0, 2.5]	1.0 [1.0, 3.0]	0.941
MLND performed			
Number of N1 stations assessed	1.0 [0.0, 2.0]	2.0 [1.0, 3.0]	<0.001
Number of N2 stations assessed	0.0 [0.0, 1.0]	1.0 [0.0, 2.0]	0.073
Number of total stations assessed	1.0 [0.0, 3.0]	3.0 [2.0, 4.0]	<0.001
Safety resection margin, cm	1.5 [1.0, 1.8]	2.0 [1.3, 2.8]	0.009
Pathological results			
Size, cm	1.10 [0.80, 1.50]	1.30 [1.00, 1.80]	0.108
Cell types			0.397
Squamous cell	0 (0.0)	3 (3.0)	
Adenocarcinoma	29 (93.5)	93 (92.1)	
Large cell	1 (3.2)	0 (0.0)	
Others	1 (3.2)	5 (5.0)	
Lymphovascular invasion	0 (0.0)	4 (4.0)	0.572
Visceral pleural invasion	1 (3.2)	3 (3.0)	1
Clinical outcome			
Chest tube dwelling time, days	3.0 [2.0, 4.0]	3.0 [2.0, 5.0]	0.215
Complication	4 (12.9)	7 (6.9)	0.285
Prolonged air leakage	2 (50.0)	4 (57.1)	
Pneumonia	1 (25.0)	1 (14.3)	
Pneumothorax	0 (0.0)	2 (28.6)	
Others	1 (25.0)	0 (0.0)	
Recurrence	1 (3.2)	0 (0.0)	0.235
Mortality	4 (12.9)	3 (3.0)	0.052
Follow‐up duration, months	36.3 [26.7, 50.7]	30.0 [23.5, 47.6]	0.225

*Note*: Data are presented as *n* (%) or median [interquartile range].

Abbreviations: LS, left lung segments; MLND, mediastinal lymph node dissection; RS, right lung segments; VATS, video‐assisted thoracoscopic surgery.

### Early outcomes, including pulmonary function

The median chest‐tube indwelling time did not differ between the two groups, and the complication rates were similar. One case of early mortality occurred 22 days after surgery; the patient underwent a nonanatomical RS2 segmentectomy and died of postoperative pneumonia (Table 2).

Three to 6 months after surgery, 127 patients had available CT images within the same period. Patients who had a lung plication of ≥5 mm on the CT images were significantly more frequently observed in the nonanatomical segmentectomy group (65.5% vs. 27.3%, *p* < 0.001) (Table 3). However, among the 88 patients with available postoperative spirometry results, changes in pulmonary function test variables were not significantly different between the two groups (Table 3).

**TABLE 3:** Changes in pulmonary function according to the quality of segmentectomy.

Factor	Segmentectomy category		*p*‐value
	Nonanatomical	Anatomical	
	*N* = 31	*N* = 101	
Postoperative plication in CT			<0.001
Plication thickness 5 mm and more	19 (65.5)	27 (27.3)	
Plication thickness under 5 mm	10 (34.5)	72 (72.7)	
Changes in pulmonary function tests			
FVC, L	−0.35 [−0.53, −0.23]	−0.31 [−0.59, −0.14]	0.651
FEV1, L	−0.31 [−0.45, −0.14]	−0.27 [−0.44, −0.11]	0.912
FEV1, %	−15.0 [−20.5, −10.0]	−10.0 [−21.0, −4.0]	0.374
FVC/FEV1 ratio, %	0.0 [−4.5, 2.5]	−3.0 [−4.0, 0.0]	0.337
DLCO, %	−17.0 [−71.0, −8.2]	−19.5 [−31.0, −12.7]	0.759
DLCO/VA, %	−3.5 [−75.7, 0.0]	−6.5 [−14.2, −2.0]	0.715

Abbreviations: CT, computed tomography; DLCO, diffusing capacity of the lung for carbon monoxide; FEV1, forced expiratory volume in one second; FVC, forced vital capacity; VA, alveolar volume.

### Long‐term oncological outcomes

**FIGURE 2:**
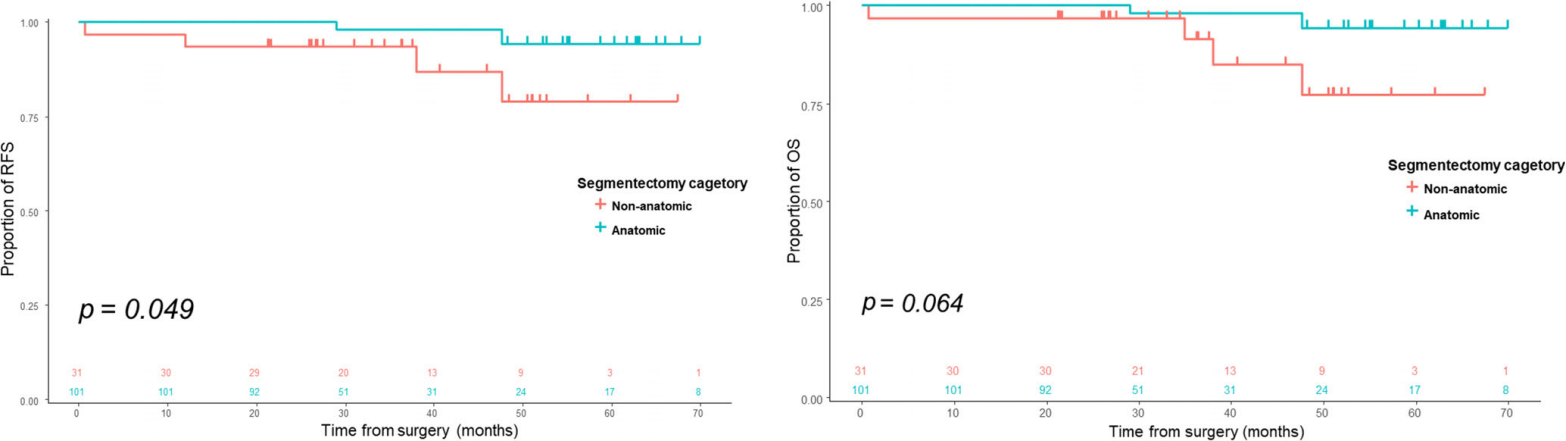
Clinical outcomes of patient with stage I non‐small cell lung cancer according to the quality of segmentectomy. OS, overall survival; RFS, recurrence‐free survival.

Compared to nonanatomical resection, anatomical segmentectomy demonstrated a better recurrence‐free survival (*p* = 0.049); however, overall survival was not statistically different between the groups (*p* = 0.064) (Figure 2). During a median follow‐up of 30.5 months, there was one case of recurrence and seven deaths. The recurrent case occurred in a 77‐year male who underwent an RS2 nonanatomical segmentectomy and was finally diagnosed with NSCLC with a large cell neuroendocrine tumor. Locoregional recurrence was observed in the right upper paratracheal lymph node 12 months after surgery, and the patient died of cancer progression at 34 months. The causes of death among the seven patients were pneumonia (4/7), cerebral hemorrhage (1/7), other malignancies (1/7), and unidentified (1/7).

Cox‐proportional hazard regression analysis for recurrence‐free and overall survival revealed that anatomical segmentectomy was a protective factor for these patients (hazard ratio [HR]: 0.15, 95% confidence interval [CI]: 0.03–0.76, *p* = 0.022; HR: 0.16, 95% CI: 0.03–0.85, *p* = 0.031) (Table 4).

**TABLE 4:** Risk factor analysis for recurrence‐free and overall survival.

A. Recurrence‐free survival				
Clinical factor	Univariate analysis		Multivariate analysis	
	Hazard ratio (95% CI)	*p*‐value	Hazard ratio (95% CI)	*p*‐value
Male (ref. female)	4.73 (0.89–25.28)	0.069	0.26 (0.01–12.1)	0.493
Age over 65	9.07 (1.09–75.62)	0.042	1.33 (0.08–20.0)	0.838
Hypertension	0.82 (0.17–3.98)	0.813		
Diabetes mellitus	2.27 (0.44–11.75)	0.332		
Smoking history (+)	11.04 (2.13–57.1)	0.004	8,85 (1.58–49.4)	0.013
CTR ≥ 0.75	5.62 (1.25–25.23)	0.024	3.62 (0.50–26.0)	0.222
FEV1/FVC	0.94 (0.87–1.01)	0.113		
Anatomical segmentectomy	0.25 (0.06–1.13)	0.072	0.15 (0.03–0.76)	0.022
Number of total MLND stations	1.26 (0.84–1.89)	0.273		
Number of N2 MLND stations	1.59 (0.97–2.6)	0.066	1.20 (0.66–2.22)	0.543
VATS approach	0.31 (0.06–1.66)	0.172		
Safety margin ≥ 2 cm	0.53 (0.06–4.59)	0.572		
Pathological size ≥ 2 cm	7.94 (1.51–41.8)	0.014	8.12 (1.36–48.7)	0.021
In‐hospital complication	6.04 (0.59–61.75)	0.132		

B. Overall survival				
Clinical factor	Univariate analysis		Multivariate analysis	
	Hazard ratio (95% CI)	*p*‐value	Hazard ratio (95% CI)	*p*‐value
Male (ref. female)	4.79 (0.9–25.6)	0.067	0.19 (0.01–15.9)	0.462
Age over 65	9.00 (1.08–75.04)	0.042	1.35 (0.08–22.0)	0.832
Hypertension	0.81 (0.17–3.91)	0.790		
Diabetes mellitus	2.37 (0.46–12.31)	0.300		
Smoking history (+)	10.92 (2.11–56.5)	0.004	8.40 (1.52–46.6)	0.014
CTR ≥ 0.75	5.59 (1.24–25.12)	0.025	3.44 (0.47–25.3)	0.225
FEV1/FVC	0.94 (0.87–1.01)	0.113		
Anatomical segmentectomy	0.27 (0.06–1.2)	0.085	0.16 (0.03–0.85)	0.031
Number of N2 MLND stations	1.25 (0.83–1.87)	0.290		
VATS approach	0.32 (0.06–1.73)	0.190		
Safety margin ≥ 2 cm	0.57 (0.07–4.87)	0.600		
Pathological size ≥ 2 cm	7.89 (1.5–41.52)	0.015	7.74 (1.31–45.8)	0.024
In‐hospital complication	7.70 (0.75–78.91)	0.086	0.56 (0.01–16.6)	0.739

Abbreviations: CI, confidence interval; CTR, consolidation tumor ratio; FEV1, forced expiratory volume in one second; FVC, forced vital capacity; MLND, mediastinal lymph node dissection; VATS, video‐assisted thoracoscopic surgery.

### Subgroup analysis for complex segmentectomies

Among patients who underwent complex segmentectomies, no differences were observed regarding hospital stay and complication rates. However, the decrease in FEV1 (%) was significantly lower in the anatomical segmentectomy group (−18.0% vs. −8.0%; *p* = 0.021). Thick plication on postoperative CT images was also observed in the nonanatomical segmentectomy group; however, the difference was not statistically significant (*p* = 0.059) (Table 5).

**TABLE 5:** Pulmonary function changes in complex segmentectomy according to the quality of segmentectomy.

Factor	Segmentectomy category		*p*‐value
	Nonanatomical	Anatomical	
	*N* = 20	*N* = 37	
Postoperative plication in CT			0.059
Plication thickness 5 mm and more	13 (68.4)	15 (41.7)	
Plication thickness under 5 mm	6 (31.6)	21 (58.3)	
Changes in pulmonary function tests			
FVC, L	−0.34 [−0.51, −0.23]	−0.31 [−0.35, −0.19]	0.377
FEV1, L	−0.33 [−0.45, −0.14]	−0.25 [−0.36, −0.11]	0.678
FEV1, %	−18.0 [−20.5, −14.0]	−8.0 [−15.5, −3.0]	0.021
FVC/FEV1 ratio, %	0.0 [−4.5, 3.5]	−3.0 [−4.0, −1.5]	0.221
DLCO, %	−17.0 [−31.5, −7.5]	−18.0 [−22.5, −14.0]	0.724
DLCO/VA, %	−3.0 [−26.50 6.0]	−5.0 [−15.50–2.50	0.438

Abbreviations: CT, computed tomography; DLCO, diffusing capacity of the lung for carbon monoxide; FEV1, forced expiratory volume in one second; FVC, forced vital capacity; VA, alveolar volume.

## DISCUSSION

This study described the importance of segmentectomy quality by comparing the patient prognosis between anatomical and nonanatomical segmentectomies. By reviewing our institution's results, we could determine how the quality of segmentectomy has changed with the accumulation of more evidence related to this procedure. Notably, anatomical segmentectomy demonstrated a significant impact, even in patients with GGO‐dominant tumors.

Division of anatomical structures should be the primary goal of segmentectomy; however, surgeons must overcome significant hurdles in their surgical skills, and technical challenges are inevitable. In this study, more nonanatomical segmentectomies were performed in patients who required complex segmentectomies, representing a significant learning curve and highlighting the technical difficulties of removing segmental vessels and bronchi. Among the major segmental structures, segmental bronchus resection was considerably more difficult than vessel resection; 29 of 31 nonanatomical segmentectomies were due to no segmental bronchus resection. However, as surgeons gained more experience, the rate of anatomical resection increased to 94% in 2020. This advancement in surgical skills has been observed in several institutions.

Anatomical segmentectomy was found to be a significant protective factor against future events, such as recurrence or mortality. As this study cohort comprised pathological stage I and GGO‐dominant tumors, it was difficult to observe more recurrent cases and compare the oncologic benefits. However, it is interesting that most mortalities occurred due to respiratory causes (4 out of 7 deaths were pneumonia‐related). It is unclear whether anatomical segmental resection is directly related to the preservation of functional reserves for future adverse events, as spirometry did not exhibit significant benefits; however, other factors, such as bronchial angulation and intrathoracic dead space, can affect the results of segmentectomy. Therefore, other assessments should be considered when measuring pulmonary function after surgery.

We believe that postoperative plication may be one of the factors explaining different patient prognoses. Lung plication, manifesting as a fibrotic linear line, is inevitable when surgeons use staplers instead of electrocauterization. This limits the full expansion of the residual lung parenchyma and exerts more tension on the lung surface, sometimes resulting in subsegmental atelectasis. Lung plication is more frequently observed when stapling is used for the intersegmental plane division^12^; however, few studies still explain the long‐term effects of compromised lung expansion due to plication.

Several studies have reported poor clinical outcomes in lung cancer patients with preoperative fibrosis^13^ or interstitial lung disease.^14^ Although it is still too early to classify lung plication as one of these pathogenic findings, plication can limit lung inflation and lead to detrimental effects when patients are exposed to other respiratory insults. Therefore, further assessment and interpretation of plications are necessary to evaluate the lasting effects after segmentectomies.

Some studies have argued that nonanatomical segmentectomies should be classified as wide‐wedge or large‐wedge resections. We agree that it is difficult to classify cases involving only parenchymal stapling as segmentectomies; however, there are many cases where only one blood vessel or bronchus was divided, which also differ from wedge resections. Owing to the inherent heterogeneity of this procedure, a clear guideline or reporting protocol for optimal segmentectomy is needed. If several criteria for the quality measure were applied, the actual acceptable rate of segmentectomy would decrease, as exhibited in the National Cancer Database study.^7^ Therefore, studies that re‐evaluate the quality of segmentectomies would be a good starting point for more transparency.

Our study had several limitations regarding the generalization of results. First, this retrospective review included a very limited number of patients; therefore, the statistical power was not optimal. Second, there was variability regarding the time at which post‐operative CTs and spirometry were obtained, which was between 3 and 6 months. Some patients could not undergo spirometry owing to precautions taken during the coronavirus disease pandemic; therefore, differences in time factors should be considered. Third, intraoperative identification of the intersegmental plane was performed using several methods. As other studies have reported, intersegmental plane detection methods other than indocyanine green injection might not be accurate and may lead to differences in resected parenchymal volume. Finally, lung plication measurement based on CT is a new concept. Further evaluation and standardization are therefore required.

In conclusion, this study evaluated the importance of anatomical segmentectomy, even in patients with early‐stage lung cancer with GGO components. Anatomical segmentectomy was associated with a lower incidence of adverse outcomes during follow‐up and postoperative lung plication was observed less frequently on radiological assessments. Institutional evaluation of the quality of segmentectomy is necessary to lower heterogeneity and improve the outcomes.

## AUTHOR CONTRIBUTIONS

Wongi Woo: Conceptualization, methodology, data curation, formal analysis, investigation, software, writing – original draft, review and editing. Jimin Lee: Data curation, writing, review and editing. Dae Hyun Jin: Methodology, data curation, writing, review and editing. Jihoon Kim: Methodology, data curation, writing, review and editing. Duk Hwan Moon: Conceptualization, methodology, validation, supervision, project administration, writing – review and editing. Sungsoo Lee: Supervision, project administration, writing – review and editing.

## CONFLICT OF INTEREST STATEMENT

The authors declare no financial or nonfinancial conflicts of interest, including funding, provision of study materials, medical writing, or article‐processing charges.

## Data Availability

The data underlying this study will be shared with the corresponding author upon request.

## References

[1] Horeweg N , van der Aalst CM , Thunnissen E , Nackaerts K , Weenink C , Groen HJM , et al. Characteristics of lung cancers detected by computer tomography screening in the randomized NELSON trial. Am J Respir Crit Care Med. 2013;187(8):848–854. 10.1164/rccm.201209-1651OC 23348977

[2] Carbone DP , Gandara DR , Antonia SJ , Zielinski C , Paz‐Ares L . Non‐small‐cell lung cancer: role of the immune system and potential for immunotherapy. J Thorac Oncol. 2015;10(7):974–984. 10.1097/JTO.0000000000000551 26134219 PMC4618296

[3] Hirsch FR , Scagliotti GV , Mulshine JL , Kwon R , Curran WJ Jr , Wu YL , et al. Lung cancer: current therapies and new targeted treatments. Lancet. 2017;389(10066):299–311. 10.1016/S0140-6736(16)30958-8 27574741

[4] Lim E , Batchelor TJ , Dunning J , et al. Impact of video‐assisted thoracoscopic lobectomy versus open lobectomy for lung cancer on recovery assessed using self‐reported physical function: VIOLET RCT. Health Technol Assess. 2022;26(48):1–162. 10.3310/THBQ1793 PMC9791462 36524582

[5] Saji H , Okada M , Tsuboi M , Nakajima R , Suzuki K , Aokage K , et al. Segmentectomy versus lobectomy in small‐sized peripheral non‐small‐cell lung cancer (JCOG0802/WJOG4607L): a multicentre, open‐label, phase 3, randomised, controlled, non‐inferiority trial. Lancet. 2022;399(10335):1607–1617. 10.1016/S0140-6736(21)02333-3 35461558

[6] Altorki N , Wang X , Kozono D , Watt C , Landrenau R , Wigle D , et al. Lobar or sublobar resection for peripheral stage IA non‐small‐cell lung cancer. N Engl J Med. 2023;388(6):489–498. 10.1056/NEJMoa2212083 36780674 PMC10036605

[7] Logan CD , Jacobs RC , Feinglass J , Lung K , Kim S , Bharat A , et al. National trends in the quality of segmentectomy for lung cancer. J Thorac Cardiovasc Surg. 2023;165(1):351–363.e20. 10.1016/j.jtcvs.2022.05.050 36088143 PMC9771936

[8] Weiss KD . When a segmentectomy is not a segmentectomy: quality assurance audit and evaluation of required elements for an anatomic segmentectomy. J Thorac Cardiovasc Surg. 2023;165(6):1919–1925. 10.1016/j.jtcvs.2022.08.042 36244821

[9] Handa Y , Tsutani Y , Mimae T , Tasaki T , Miyata Y , Okada M . Surgical outcomes of complex versus simple segmentectomy for stage I non‐small cell lung cancer. Ann Thorac Surg. 2019;107(4):1032–1039. 10.1016/j.athoracsur.2018.11.018 30550801

[10] Okubo Y , Yoshida Y , Yotsukura M , Nakagawa K , Watanabe SI . Complex segmentectomy is not a complex procedure relative to simple segmentectomy. Eur J Cardiothorac Surg. 2021;61(1):100–107. 10.1093/ejcts/ezab367 34355732

[11] Handa Y , Tsutani Y , Mimae T , Miyata Y , Okada M . Complex segmentectomy in the treatment of stage IA non‐small‐cell lung cancer. Eur J Cardiothorac Surg. 2020;57(1):114–121. 10.1093/ejcts/ezz185 31230086

[12] Matsumoto M , Shirahashi K , Yamamoto H , Miyamaoto Y , Komuro H , Doi K , et al. Division of the intersegmental plane using electrocautery for segmentectomy in clinical stage I non‐small cell lung cancer. J Thorac Dis. 2018;10(Suppl 10):S1215–S1221. 10.21037/jtd.2018.03.65 29785296 PMC5949397

[13] Garner M , Taylor M , Smith M , Abah U , Shackcloth M , Granato F , et al. Pre‐existing pulmonary fibrosis is associated with adverse outcomes after lung resection. Respir Med. 2022;205:107037. 10.1016/j.rmed.2022.107037 36347082

[14] Ki MS , Kim SY , Kim EY , Jung JY , Kang YA , Park MS , et al. Clinical outcomes and prognosis of patients with interstitial lung disease undergoing lung cancer surgery: a propensity score matching study. Clin Lung Cancer. 2023;24(1):e27–e38. 10.1016/j.cllc.2022.10.003 36376171

